# Human indole(ethyl)amine-N-methyltransferase (hINMT) catalyzed methylation of tryptamine, dimethylsulfide and dimethylselenide is enhanced under reducing conditions - A comparison between 254C and 254F, two common hINMT variants

**DOI:** 10.1371/journal.pone.0219664

**Published:** 2019-07-16

**Authors:** Brian Torres, James S. Tyler, Kenneth A. Satyshur, Arnold E. Ruoho

**Affiliations:** 1 Department of Neuroscience, School of Medicine and Public Health, University of Wisconsin, Madison, Wisconsin, United States of America; 2 Small Molecule Screening Facility, Carbone Cancer Center, School of Medicine and Public Health, University of Wisconsin, Madison, Wisconsin, United States of America; Weizmann Institute of Science, ISRAEL

## Abstract

Phenylalanine and cysteine comprise common miss-sense variants (i.e., single nucleotide polymorphisms [SNPs]) at amino acid position 254 of the human indole(ethyl)amine-N-methyltransferase (hINMT). The phenylalanine variant, which occurs in linkage disequilibrium with two 3’ UTR SNPs, has been reported to associate with elevated urine levels of trimethylselenonium (TMSe), the Se-methylated product of volatile dimethylselenide. hINMT allozymes expressing either cysteine (254C) or phenylalanine (254F) at position 254 were compared for enzyme activity (i.e., K_m_ and V_max_) towards the INMT substrates tryptamine, dimethylsulfide (DMS) and dimethylselenide (DMSe) *in vitro*. The SNP 254C had a higher V_max_ for DMS and tryptamine in the presence of reducing agent than in its absence. Conversely, V_max_ for 254F was insensitive to the presence or absence of reducing agent for these substrates. SNP 254F showed a lower K_m_ for tryptamine in the absence of reducing agent than 254C. No statistically significant difference in V_max_ or K_m_ was observed between 254C and 254F allozymes in the presence of reducing agent for DMSe, The K_m_ values for DMSe methylation were about 10-fold (254C) or 6-fold (254F) more favorable than for tryptamine methylation with reducing agent present. These findings indicated that: 1) That phenylalanine at position 254 renders hINMT methylation of substrates DMS and tryptamine insensitive to a non reducing environment. 2) That human INMT harbors significant thioether-S-methyltransferase (TEMT) activity with a higher affinity for DMSe than tryptamine, 3) The reduction of a 44C/254C disulfide bond in hINMT that increases V_max_ is proposed.

## Introduction

Methyltransferases catalyze the transfer of a methyl group from the donor, S-adenosyl-L- methionine (SAM), to acceptor molecules. Indole(ethyl)amine-N-methyl transferase (INMT) is a soluble 263 amino acid Class 1 “small-molecule” methyl transferase, containing 11 cysteines with a molecular weight of approximately 25 kilodaltons. INMT was initially identified by Axelrod as an enzyme that methylates tryptamine to N,N-dimethyltryptamine (DMT) in rabbit lung homogenates [1]. Cloning and characterization of rabbit INMT confirmed a preference of the enzyme for indole(ethyl)amine-directed N-methylation relative to structurally similar compounds such as the biogenic amines [2, 3]. Cloning has indicated a close sequence homology (59%) to mouse thioether-S-methyltransferase (mTEMT). mTEMT has been shown to methylate volatile dimethylsulfide (DMS), dimethylselenide (DMSe) and dimethyltelluride (DMTe). Elevated levels of these volatile compounds can lead to expiration from the lung and skin, and a garlic-odored breath associated with selenium (Se) toxicity (selenosis) and tellurium toxicity [4–7]. Methylation of DMS, DMSe and DMTe yields the positively charged non volatile trimethylated products trimethylsulfonium (TMS), trimethylselenonium (TMSe) and trimethyltelleronium (TMTe), essential for urinary excretion [8–10]. Recent work has shown that human INMT (hINMT) can methylate dialkylthiols [11]. The INMT enzyme has been shown to occur in peripheral primate tissues such as the pineal gland, retinal ganglion cells and spinal ventral horn motor neurons [12].

Several common INMT miss-sense single nucleotide polymorphisms (SNPs) with minor allele frequencies (MAFs) greater than 0.05 have been reported including, D28N, M206V, G219E and C254F (variation viewer, NCBI) [13, 14] and a rare miss-sense SNP, H46P, has been reported to associate with Hirschprung’s Disease [15] (S2 Fig). To assess by SDS gel analysis any obvious biochemical features of these INMT SNPs, the SNPs were *E*. *Coli* expressed and purified to homogeneity INMT SNPs, 254C, 254F, D28N, H46P, M206V and N245S (S3 Fig). Two hINMT isoforms have been identified. Isoform 1 (NP_006765.4) encodes a glycine at position 52 and is 263 amino acids in length whereas isoform 2 lacks this glycine and is 262 AAs in length. Data obtained from isoform 1 is presented in this study. Recently it was reported that individuals with elevated urine-TMSe (U-TMSe) levels, i.e., ‘producers’, were more likely to express phenylalanine (seq: TTC) than cysteine (seq: TGC) at amino acid position 254 of the protein (C254F; rs4720015; pos. 30,795,436; GRCh37.p13; S2 Fig) [9, 16]. The phenylalanine-encoding version of this variant occurs in linkage disequilibrium with two 3’ UTR INMT SNPs (rs6970396; [A; pos. 30,795,547] and rs1061644 [T; pos. 30,796,530]; S2 Fig). *In silico* evidence suggests that the 3’ UTR variant nucleotides in linkage disequilibrium with the T nucleotide encoding protein position 254 (i.e., phenylalanine) may alter INMT expression levels [9, 14]. Therefore, given the likelihood for multiple influences affecting ultimate INMT activity levels, we sought to isolate the contribution, if any, of the cysteine vs. phenylalanine variation at position 254 on INMT enzyme activity (i.e.; K_m_, V_max_ values) on TMSe production *in vitro*. We compared the methylation activities of INMT expressing either cysteine (254C) or phenylalanine (254F) towards tryptamine, DMS and DMSe (methylation reactions S1 Fig).

## Materials and methods

### *E. coli* expression and purification of hINMT proteins (254C, 254F, D28N, H46P, M206V, N245S)

N-terminally His-tagged human INMT proteins were expressed, amplified and batch-purified using standard purification procedures in the absence of reducing agent. Miss-sense mutations were created using the Quickchange method (Aligent) on pet28a vector encoding ‘WT’ (254C) hINMT (S2 Fig) and verified by sequencing. All hINMT genes were N-terminally His-tagged to enable cobalt bead purification. E-coli BL-21 (Lucigen, Madison) cells were transformed with pet28a vector encoding N-terminally His-tagged hINMT construct. Following pre-culture, transformed BL-21 cells were grown to OD_600_ = 600 at 37° in 6 L Luria broth (LB) under kanamycin (50 μg/ml) selection at which point hINMT expression was induced with 1 mM isopropyl β-D-1-thiogalactopyranoside (IPTG) and the cultures grown an additional 3 hours. Bacteria were collected by centrifugation and resuspended in 25 ml buffer A (50 mM tris-HCl pH 7.5, 150 mM NaCl, protease inhibitor cocktail [Dot Scientific, Burton]). The suspension was sonicated eight times in 30 sec. on/off cycles at 4°C at output 4. Sonicated membranes were pelleted by centrifugation (13,000 x G, 20 min). 500 μl of buffer A pre-equilibrated HisPure cobalt resin (ThermoFisher Scientific, Waltham) and 20 mM imidazole were added to the lysate and rotated for 1 hr at ambient temperature. The resin was washed 3X in 2 ml buffer B (50 mM NaH_2_PO_4_ pH 7.8, 20 mM imidazole) and eluted with 2X 3 ml buffer B incubations with 300 mM imidazole. Eluted volumes were combined and dialyzed twice overnight in 6 L of buffer B at 4°C to remove imidazole. The dialyzed proteins were concentrated to approximately 1 mg/ml using a Vivaspin 6 column (Sartorius, Goettingen) and verified by acrylamide gel Coomassie staining for molecular size and purity. Pure protein yield was generally in the 2–3 mg range. Protein was adjusted to a final concentration of ca. 0.5 mg/ml and stored in 50–90 μl aliquots at -80°C in 20% glycerol, 40 mM NaH_2_PO_4_ pH 7.5; 130 mM NaCl until use. For added comparison, pure protein samples of the hINMT allozymes WT (254C), D28N, H46P, M206V, G219E and 254F are shown in the presence of reducing agent in S3 Fig.

### hINMT assays: Tryptamine, DMS, DMSe methylations

Assays of hINMT activity were conducted as previously described [17] in tightly sealed 2 ml Eppendorf tubes. For N-methyltryptamine (NMT) and N,N-dimethyltryptamine (DMT) synthesis, 5 μg of -80°C frozen hINMT protein was combined with 35 μl 20 mM NaH_2_PO_4_ pH 7.9 and tryptamine (Sigma-Aldrich; Tryptamine HCl; ≥99% purity) scaled to indicated final concentrations in a final reaction volume of 50 μl. For TMSe and TMS synthesis, 10 μg of protein was combined with 35 μl 50 mM bis-tris propane (Sigma-Aldrich) pH 7 and scaled for DMSe or DMS (Sigma-Aldrich; Dimethyl selenide, Dimethyl sulfide; ≥99% purity) methylation to a final reaction volume of 50 μl. DMSe was kept continually at 4°C and stored under argon after each use. To all reactions, 5 μl ^14^C S-adenosyl-L-methionine (SAM; Perkin Elmer, specific activity 0.165 mCi/mM) was added to yield a 35 μM final concentration. 0.5 μl of 100X (1.5 M) dithiothreitol (DTT) was added as indicated. Buffers for conditions with reduced-glutathione were pH pre-adjusted to account for glutathione (Sigma-Aldrich) -induced acidity. Reactions were adjusted to 50 μl final volume with water and incubated at 30°C for 60 min (tryptamine methylation) or 90 min (DMSe and DMS methylation). Reactions were terminated by addition of a total of 300 μl (0.5 M) potassium tetraborate (Alfa Aesar) with (TMS and TMSe assays) or without (NMT/DMT assays) 0.14 M heptane sulfonic acid (Chem-Impex) followed by brief vortexing. The ^14^C methylated products was extracted by addition of 1 ml 60:40 v/v (for TMS or TMSe) or 3:97 v/v (for NMT/DMT) of isoamyl alcohol:toluene, vortexed followed by a two minute 14,000 rpm microfuge spin. Seven hundred μl of the top organic layer was added to 5 ml scintillation fluid in counting vials. Radioactivity was assessed with a Perkin Elmer scintillation counter for 2 min. Total ^14^C radiolabelled NMT/DMT was identified as previously reported [17]; TMS was previously identified by Mozzier et al., [18] and TMSe was identified by chromatographic co-migration with authentic nonradioactive TMSe. Unless otherwise indicated, experiments were all performed a minimum of two times with duplicate assessments for each concentration of analyzed. V_max_ (fm product/[μg∙min]) and K_m_ (mM) values were obtained using the Michaelis-Menten function of GraphPad Prism.

### Assessment of DMSe purity

Because DMSe can readily share its bonding electrons with oxygen, possible oxidation of DMSe to DMSeO (dimethylselenoxide) and DMSeO_2_ (dimethylselenone) was assessed to be sure that the DMSe used in these experiments was pure. Infrared spectra were obtained using a Bruker Equinox 55 IR spectrophotometer. A PIKE Technologies MIRacle ATR accessory equipped with a single reflection Zinc Selenide (ZnSe) ATR crystal was used for the analyses. The results are shown in S6 Fig.

### *In silico* energy minimization of the hINMT structure

The hINMT crystal structure was cleaned of water molecules and ions in the modeling program Sybyl and hydrogens were added. S-adenosyl-L-methionine was included in the structure. Cys 254 was torsioned to place the side chain sulfur in proximity to the Cys 44 side chain sulfur. The distance was measured as 2.7 A. A bond was created between the sulfur atoms after removal of the two hydrogens. The whole molecule was energy minimized for 100 cycles using the Tripos force field. There were no major rearrangements of the backbone around the cysteines. The resultant sulfur-sulfur distance was determined to be 1.9 A.

### Protein analysis by SDS-Page

For gel analysis of INMT proteins, 5 μg of purified INMT protein sample was separated on 12% acrylamide low-cross-linked separating gels (0.06% bis-acrylamide) in 5X sample buffer with or without 10 mM DTT. Following band separation, gels were stained for 10 min in Coomassie staining buffer (50% MeOH, 10% acetic acid, 0.1% brilliant blue dye) followed by 3 X 10 min. washes in destain buffer (40% MeOH, 10% acetic acid). A final wash was allowed to extend overnight after which the staining and wash procedures were repeated once with the final wash extended until a clear resolution of all protein bands was achieved.

## Results

**Fig 1:**
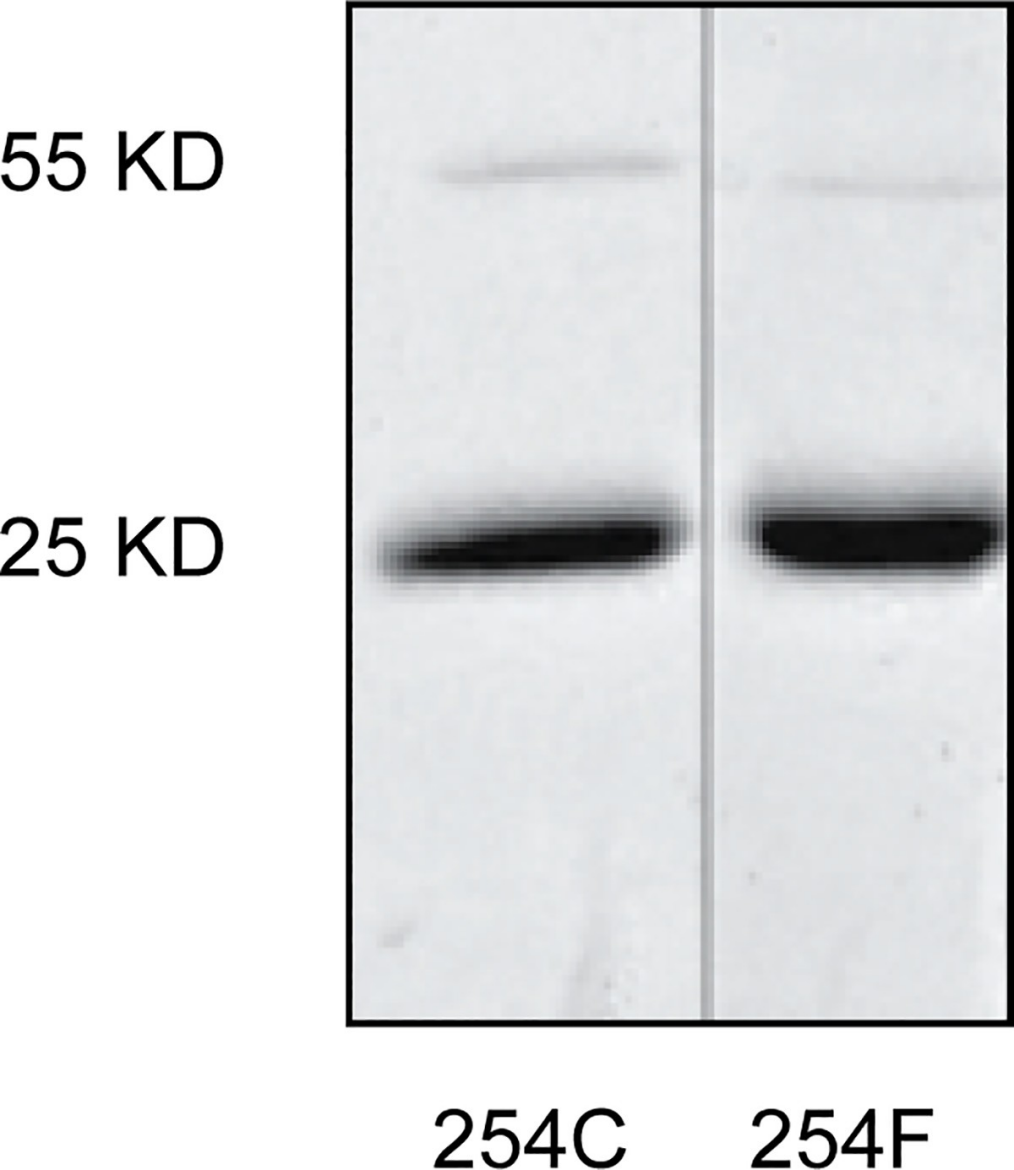
hINMT purified protein. Coomassie stained SDS-PAGE of 254C and 254F allozymes used in the kinetic study indicating the purity and the apparent molecular size.

**Fig 2:**
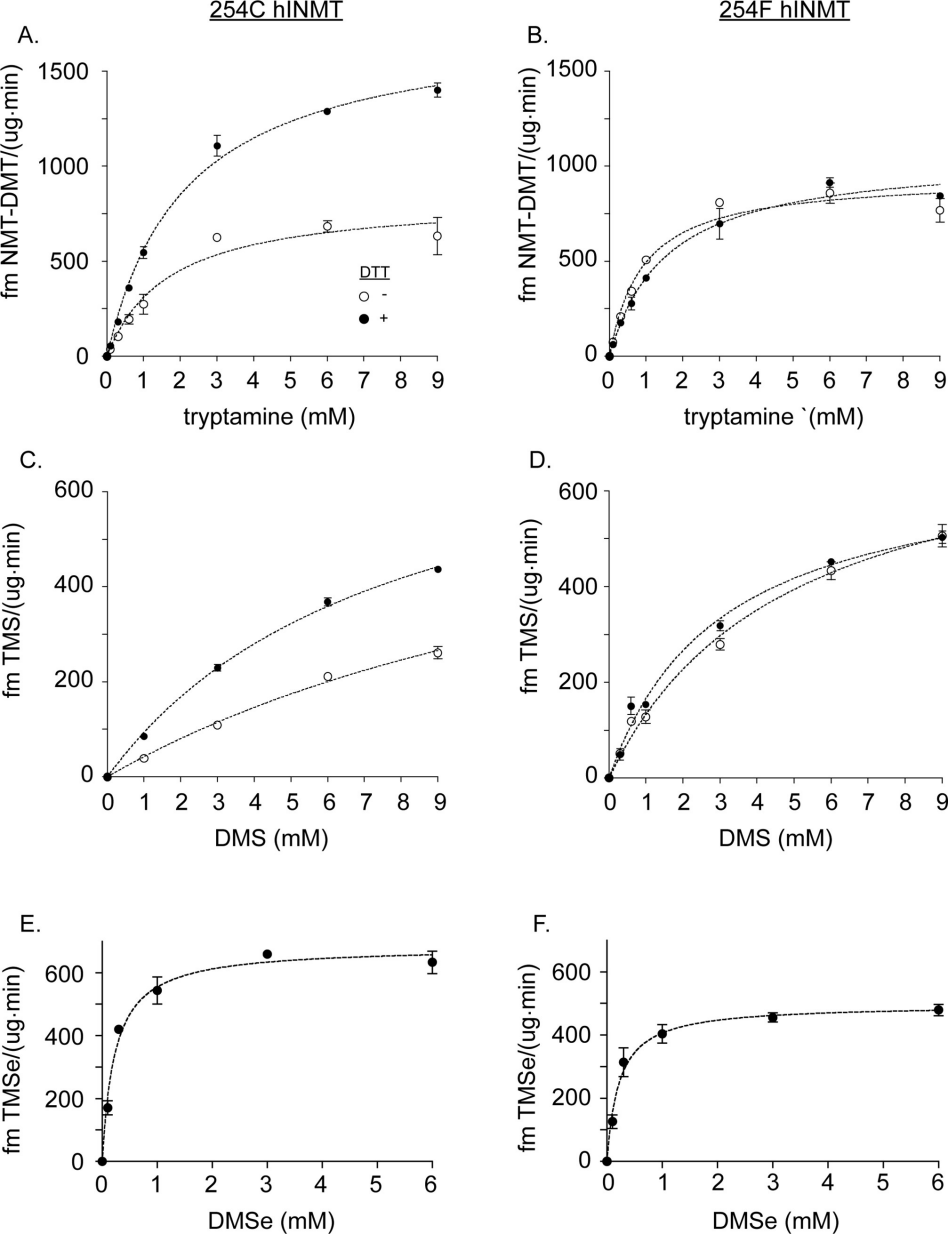
hINMT enzyme activity: 254C vs. 254F. Comparison of hINMT-catalyzed methylated product formation for 254C (A,C,E) and 254F (B, D, F) (-/+) 15 mM DTT for the substrates tryptamine (A, B), and DMS (C,D). The results for DMSe (E,F) are shown with (+) DTT only. Indicated concentrations of substrates were co-incubated with 5 μg of 254C hINMT or 254F hINMT and 30 μM ^14-^C S-adenosyl-methionine as described in Materials and Methods. Statistical analysis of results is described in Table 1.

Production of methylated products from three substrates (tryptamine, DMS and DMSe) was compared between the 254C and 254F hINMT allozymes. The allozymes were expressed and purified using *E*. *coli* expression techniques with SDS-PAGE confirming expression of the expected 25 kD molecular weight (Methods; Fig 1; S3 Fig). In addition to 254C, hINMT has numerous potentially reactive cysteine residues, the thiols of which could engage in oxidation-reduction reactions with each other and potentially with substrates affecting allozyme activities [19, 20]. Production of NMT/DMT, TMS and TMSe was compared (-/+) 15 mM DTT (similar data to 15 mM DTT was obtained with tryptamine and 2, 5 or 10 mM DTT; S4A Fig). DTT was chosen because of its relative pH neutrality, lack of bulk, and possession of two reducing equivalents per molecule. Representative data for product formation are shown in Fig 2. A comparison of K_m_ and V_max_ values for product formation by the 254C and 254F allozymes is shown in Table 1.

**Table 1:** K_m_ (mM) and V_max_ (fm product/[μg∙min]) values for methylated product formation: 254C vs 254F hINMT*.

			K_m_			V_max_		
substrate	allozyme	DTT	ave	SD	*-/+DTT*: *p val*^†^, *n*	ave	SD	*-/+DTT*: *p value*, *n*
tryptamine	254C	-	1.70	.05		.73	.14	
		+	2.91	1.09	*0*.*300*, *2*	1.74	0.04	*0*.*0114*, *2*
	254F	-	0.93	0.04		1.02	0.11	
		+	1.64	0.13	*0*.*0305*, *2*	1.09	0.05	*0*.*338*, *2*
DMS	254C	-	0.81	0.16		5.39	0.43	
		+	0.89	0.15	*0*.*133*, *2*	16.69	1.41	*0*.*0015*, *2*
	254F	-	0.63	0.20		3.12	2.31	
		+	0.62	0.07	*0*.*056*, *2*	2.21	1.16	*0*.*404*, *2*
DMSe	254C	-	ND**	ND		ND	ND	
		+	0.24	0.13	*nd*, *3*	0.61	0.13	*nd*, *3*
	254F	-	ND	ND		ND	ND	
		+	0.26	0.03	*nd*, *3*	0.48	0.15	*nd*, *3*

*, * * values calculated from minimum two assays with duplicate points, ND, not determined

^†^Significant difference between (-/+) DTT values for K_m_ and V_max_ parameters.

Unpaired t-test comparisons were carried out between the (-/+) DTT values obtained for each substrate/allozyme combination. For example, 254C tryptamine V_max_ (-) DTT (0.73 ± 0.14) was compared by unpaired t-test to the respective 254C (+) DTT condition (1.74 ± 0.04) resulting in a p value of 0.0114. A significant difference was the result of a P value < 0.05.

1. Tryptamine as substrate (Fig 2A and 2B; Table 1)
  - Without DTT, the K_m_ was lower for 254F than 254C (0.93 fmoles product/[μg∙min] vs 1.7 fmoles product/[μg∙min]; unpaired t-test p value = 0.0027) but no change in V_max_ was observed. With DTT, no difference was observed between 254C and 254F K_m_ values. However, an increase in V_max_ value occurred for 254C compared to 254F (1.74 fmoles product/[μg∙min] vs 1.09 fmoles product/[μg∙min]; unpaired t-test p value = 0.0056).
  - 254C and 254F allozymes were then compared to determine the extent to which activities were *individually* affected by the absence or presence of DTT. For 254C, the K_m_ showed no significant difference (-/+) DTT. However, a difference in V_max_ occurred for 254C: 0.81 fmoles product/[μg∙min] (-) DTT vs 1.69 fmoles product/[μg∙min] (+) DTT. For 254F, a reduction in K_m_ value but no difference in V_max_ was observed (-/+) DTT. Therefore, the V_max_ of 254C but not 254F was substantially increased by the presence of DTT.
2. DMS as substrate (Fig 2C and 2D; Table 1)
  - (-) DTT, TMS production showed no statistical difference in K_m_ or V_max_ between 254C and 254F. (+) DTT, no significant difference in K_m_ occurred between 254C and 254F. A greater V_max_ was achieved by 254C compared to 254F (16.7 fmoles product/[μg∙min] vs 2.21 fmoles product/[μg∙min]; Table 1). This 8-fold difference in V_max_ may have been influenced by the inherent volatility of DMS (see Discussion).
  - K_m_ values for 254C and 254F *individually* did not vary (-/+) DTT. However, similar to NMT/DMT production, TMS V_max_ values varied considerably between allozymes (-/+) DTT: For 254C, removal of DTT led to a substantial lowering of V_max_ (16.7 fmoles product/[μg∙min] vs 5.4 fmoles product/[μg∙min]). No V_max_ change was observed for 254F, indicating that, like NMT/DMT, TMS production by 254F was largely insensitive to the presence or absence of DTT (Fig 2D; Table 1).
3. DMSe as substrate (Fig 2E and 2F; Table 1)
  - Without DTT, the addition of DMSe resulted in denaturation/alteration of both the 245C and the 254F forms of hINMT. This was shown as inactivation of the enzyme activity and by SDS PAGE analysis (S5 Fig). As seen in S5C Fig, lane 4, (-) DTT both the 254C and the 254F enzyme forms showed bands that migrated below the position of the native enzyme when DMSe was present. This result was not observed for DMS or tryptamine (S5C Fig, lanes 2 and 3). Thus even though the starting DMSe was shown to be >99% pure (S6 Fig) formation of oxidized products (e.g., DMSeO and/or DMSeO_2_) appears to have occurred *during incubation* with the enzyme for 90 minutes at 30°C in aqueous buffer (Methods). Based on these data it was not possible to unequivocally establish the true concentrations of DMSe (-) DTT during incubations and assessment of the K_m_ and V_max_ of the two allozymes for DMSe (-) DTT was not possible.
  - With DTT, however, denaturation/alteration of 254C and 254F was completely protected (S6C Fig, lane 4 vs 8) as occurred for tryptamine and DMS regardless of the presence of DTT (S6C Fig, lanes 2/3 and 6/7. Under these reducing conditions no significant variation in K_m_ or V_max_ was observed between 254C and 254F (Fig 2E and 2F; Table 1). Therefore, with DTT present the oxidized forms of DMSe appear not to have formed during the incubations. The K_m_ was found to be approximately 10X better than that for tryptamine and the V_max_ was 1/2 to 1/3 that of tryptamine (Table 1).

In addition to the 254F hINMT variant, which occurs with moderate frequency in the general population [16], additional higher frequency miss-sense hINMT SNPs with MAFs > 0.05 include D28N, M206V and G219E (S2 Fig) [14, 21]. No human-health phenotypes have been reported to associate with these additional higher frequency miss-sense hINMT SNPs. Only one other hINMT SNP has been reported to correlate with a human-health condition; that is, a recent genome wide association study comparing Hirschsprung’s Disease patients with controls found that only the rare hINMT SNP H46P correlated with the occurrence of Hirschsprung’s Disease in affected individuals [15]. Assays of H46P hINMT activity were conducted to determine if this SNP could be correlated with an effect on enzyme activity. Attempts to assay purified H46P allozyme resulted in no detectable NMT/DMT or TMSe formation in the presence of DTT (S7 Fig). Additionally, H46P, relative to the other allozymes tested herein, migrated on SDS-PAGE at a position consistent with a dimer even with 15mM DTT present (S3 Fig).

Reduced-glutathione possesses only one reducing equivalent per molecule, is more bulky and is highly acidic. Because reduced glutathione, naturally present at millimolar cellular concentrations, maintains the redox state of intracellular protein thiol groups [20, 22], it was tested in lieu of DTT. The data showed that 254C hINMT activity did not differ substantially between the presence of reduced glutathione or DTT (S4A and S4B Fig).

## Discussion

This investigation of hINMT enzyme activity revealed two features of hINMT function: 1) cysteine at position 254 functions as a redox-sensor influencing INMT function; that is, replacement of cysteine with phenylalanine eliminated (DMS and tryptamine) sensitivity of INMT activity (measured as V_max_) to DTT. 2) 10- and 6-fold lower K_m_ values were obtained for DMSe vs tryptamine for 254C and 254F allozymes respectively in the presence of DTT (Table 1). The higher (mM) tryptamine K_m_ value is consistent with previous observations of K_m_ values for tryptamine obtained in purified INMT assays as well as assays conducted in rabbit lung homogenates under reducing conditions [1–3, 17]. The finding of a lower K_m_ for DMSe relative to tryptamine (+) DTT supports the assertion that DMSe may be preferred as a substrate over tryptamine in some tissues including the lung. The inherent volatility of DMS (boiling point 37°C) in the assays may have altered the observed K_m_ and V_max_ values for this substrate (Table 1), even though the incubation tubes were tightly sealed (see Methods), underscoring the shallower concentration profiles arising from the use of DMS, compared to DMSe or tryptamine as substrates (Fig 2). Thus, while the use of DMS as a substrate demonstrated clear differences in TMS formation between the presence and absence of DTT, for 254C but not 254F hINMT, a definite conclusion regarding the preference of DMS as a substrate relative to DMSe or tryptamine must await further investigation.

The 254C (TGC) and 254F (TTC) forms are documented as major and minor hINMT allele variants respectively, with T frequencies ranging from 0.45 (African) to 0.15 (East Asian) to 0.10 (European) [16]. A recent study demonstrated elevated frequencies of the 254F SNP compared to 254C in Bangladeshi vs. Andean populations of women with high and low mean U-TMSe levels respectively [9] providing a rationale for testing 254C vs 254F TMSe production in the present study. However, attempts to assay DMSe in the *absence* of DTT resulted in a severe reduction in hINMT activity for *both* 254C and 254F allozymes (S5A and S5B Fig) and a severe alteration of the hINMT protein appearing as a second diffuse band underneath the original hINMT band (by SDS-PAGE) not present for DMS or tryptamine (S5C Fig,lanes 2&3). These results indicated 1) that INMT was denatured/altered by DMSe oxidation products (-) DTT that formed during incubation with the enzyme regardless of C or F at position 254 and/or 2) that the DMSe concentration (-) DTT was altered to significantly reduce the DMSe concentration. Since DMSe exists in a reduced (II-) [23] state, it is unlikely that it has the potential to inflict denaturation of proteins. Fourier transform infrared spectroscopy (FTIR) performed on the DMSe used for these experiments was pure and devoid of detectable oxidized by products such as DMSeO and/or DMSeO_2_ prior to usage in the methylation assay (S6A–S6C Fig) [24–26]. It is therefore likely that the decreased production of TMSe in the absence of DTT occurred via formation of oxidized DMSe-products *during* the 90 minute methylation incubation. Thus, the findings of *lower* K_m_ and V_max_ values for DMSe-methylation compared to tryptamine-methylation appear to be relevant (Fig 2; Table 1).

**Fig 3:**
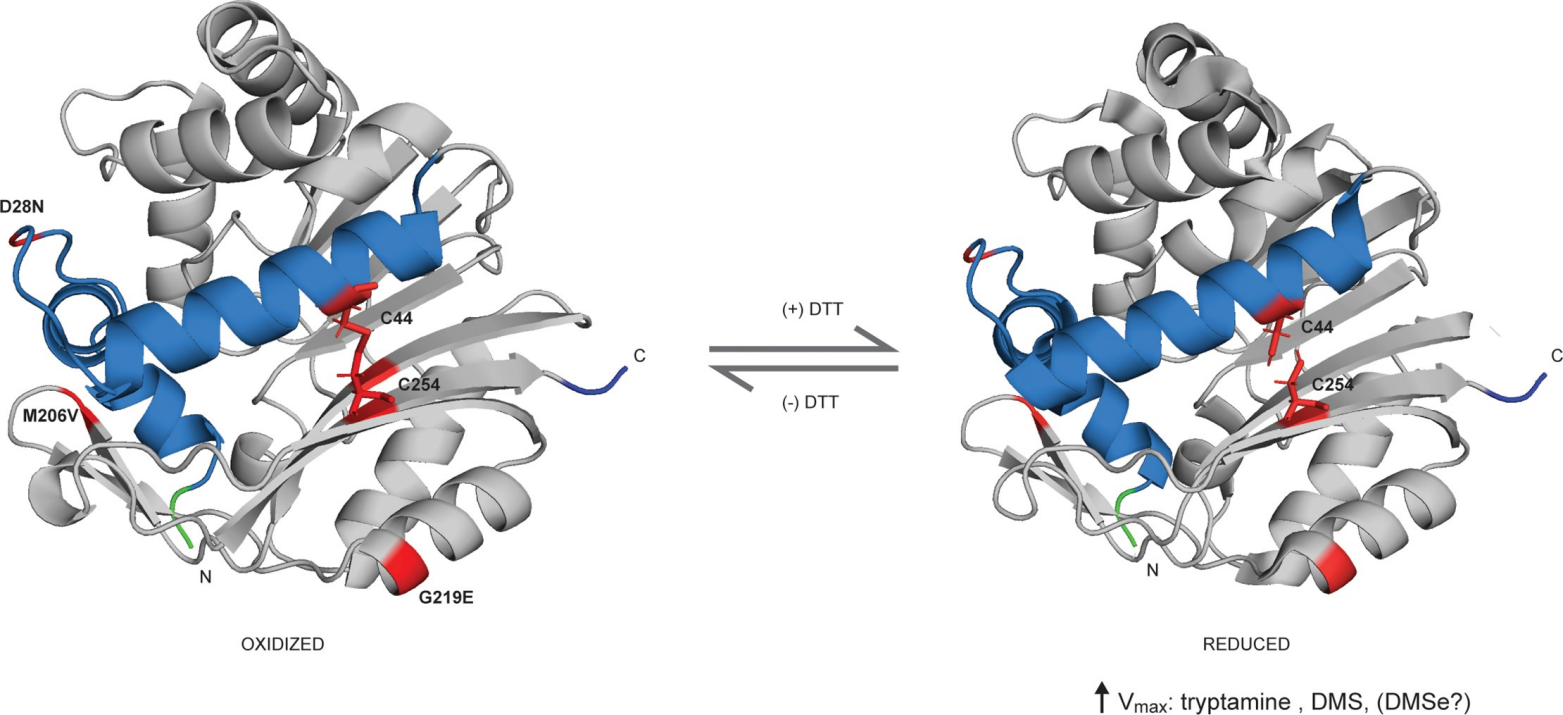
Cleavage of the proposed disulfide bond, 44C/254C, by DTT in hINMT. Left side: hINMT crystal structure prepared without reducing agent with the position of common miss-sense SNPs (D28N, M206V, G219E and C254 [note: C44 is *not* at a position of a common SNP]) red-highlighted. Right side: Reducing agent-induced cleavage of proposed disulfide bond causes an increase in 254C enzyme activity; N-terminal tandem alpha-helical regions blue-highlighted; N- and C-termini, green and blue highlighted respectively.

Several cysteine residues are present in hINMT that potentially could engage in redox reactions affecting enzyme activity [19, 20, 27, 28]. Nevertheless, when tryptamine and DMS were used, sensitivity to the presence of DTT, as determined by V_max_, occurred only when cysteine was present at position 254, indicating that replacement of this cysteine alone was sufficient to eliminate DTT-sensitivity of INMT for these substrates. This observation raises the question as to how cysteine at position 254 could confer sensitivity to reducing-agent on the enzyme. The crystal structure of INMT is available [29]. Examination of the hINMT structure shows that C254 is in proximity to C44 in the second alpha helix N-terminal region (blue region, Fig 3). *In silico* modeling supported formation of a disulfide bond between C254 and C44: energy minimization of the hINMT crystal structure using the program Sybyl resulted in a C254-C44 S-S distance of 1.9A (see Methods), consistent with the standard S-S distance of 1.8A. The data presented here is consistent with the fact that the higher V_max_ values observed for 254C hINMT product formation in the presence of DTT, compared to values obtained in the absence of DTT, arose when this proposed C254-C44 disulfide bond was cleaved by DTT (right hand side, Fig 3). It also follows that absence of the disulfide in the 254F SNP resulted in the observed insensitivity to DTT (Fig 2). We previously found evidence that the loop residues between the two tandem alpha-helix regions in the distal N-terminal region of INMT, interacted with product (DMT) or ligand (PDAT) to allosterically regulate INMT V_max_ (blue region, Fig 3) [17]. The present findings further support regulation of hINMT activity by the N-terminal region through a proposed redox-sensitive disulfide bridge connecting N-and C-terminal regions of the 254C INMT molecule. Further studies will be necessary to confirm the existence of this putative disulfide bond.

254C showed greater production of NMT/DMT and TMS compared to 245F as measured by elevated V_max_ values (Table 1). However, *in silico* evidence suggests that the higher activity of 254C in the presence of DTT may be mitigated by altered expression (due to 3’ UTR SNPs in the hINMT gene in linkage disequilibrium with the coding SNP) [9, 14]. Thus, it will be important to confirm the actual effect of these 3’ UTR variants on INMT expression *in vitro* and *in vivo*. The ability of the 254C to sense the oxidation state may be advantageous. In an oxidized environment, the formation of a disulfide bridge between opposing halves of the protein may protect the INMT molecule from denaturation/alteration by oxidants. The increased bulkiness of phenylalanine substitution in 254F appears to have interfered with access of the bulkier tryptamine molecule to the INMT active site, resulting in a lowered V_max_ for tryptamine compared to 254C, (+) DTT. However, 254F also showed a more favorable K_m_ for tryptamine, (-) DTT, supporting an elevated affinity of the 254F active site for tryptamine, raising the possibility that in severely oxidized cellular environments, formation of NMT/DMT is increased at lower tryptamine concentrations for 254F carriers. The DMT-targeted Sigma-1 receptor, an ER-chaperone protein and recently proposed heat-shock family member, undergoes activation via de-oligomerization upon agonist-interaction. Interestingly, the Sigma-1 receptor has been shown to co-localize with INMT in dorsal root ganglia, potentially placing it in a position to provide ‘first-aid’ for oxidative-damage of INMT [12, 30–34].

In conclusion, human INMT favors a reducing environment for efficient methylating enzyme activity (V_max_) as shown *in vitro*. This is not only true for S-methylation of DMSe to TMSe but also for S-methylation of DMS to TMS and N-methylation of tryptamine to NMT/DMT. Further, the K_m_ for methylation of DMSe is approximately 10-fold more favorable for the wild type enzyme under reducing conditions and/or for the 254F SNP than for tryptamine (Table 1, Fig 2) indicating preference for DMSe as a substrate compared to tryptamine. The 254F SNP abrogated the need for a reducing environment for tryptamine and DMS. These observations taken together support a role for the 254F SNP in the high TMSe producers observed previously in two human populations [9]. The fact that human INMT is promiscuous in its recognition of substrates such as dialkyl sulfur and Se compounds, as well as indole(ethyl)amines, indicates that the biological role(s) of indole(ethyl)amine-N-methyl transferase in mammalian organs may be more expansive than previously suspected. It is noteworthy that identification of INMT enzyme activity originally reported by Axelrod in rodent lung may be due to its inherent thioether-S-methyltransferase (TEMT) activity for the detoxification of volatile dialkyl sulfur and Se compounds and perhaps in the methylation of additional, as yet to be discovered, endogenous compounds.

## Conclusions

1. 254C (WT) hINMT activity was significantly affected by the presence or absence of reducing agent *in vitro*. A common variant, 254F, eliminated sensitivity to this variable for tryptamine and DMS. A 44C/254C redox sensitive disulfide bond in hINMT is proposed.
2. hINMT has significant thioether-S-methyltransferase activity and showed a more favorable K_m_ for dimethylselenide than tryptamine *in vitro* in the presence of the reducing agent DTT.

## Supporting information

S1 Fig hINMT catalyzed methylation reactions. (PDF)

S2 Fig hINMT SNP diagram. Human INMT gene SNPs referred to in this report. Chromosome number and region are indicated. “WT” refers to the profile of SNPs in a recombinant INMT construct referred to as ‘254C’ in this report. Italicized SNPs are in linkage disequilibrium with each other. SNPs indicate amino acids in the grey exon region and nucleotides in the pink 3’UTR region with the minor allele (according to 1000 Genomes Project [refer to text for details]) underlined and frequency indicated (MAF). Position refers to the chromosomal position on GRCh37.p13.
(TIF)

S3 Fig SDS-PAGE banding patterns of hINMT SNPs (+DTT 15mM). (TIF)

S4 Fig Glutathione/DTT comparison: hINMT activity. A. Comparison of hINMT-catalyzed methylated of NMT/DMT formation by 254C hINMT for tryptamine in the presence of 15 mM DTT and 15 mM reduced glutathione (GSH). Indicated concentrations of tryptamine were co-incubated with 5 μg of 254C hINMT and 30 μM ^14^C S-adenosyl-L-methionine as described in Materials and Methods. Each bar represents an experiment performed with duplicate values with p values (two-tailed t-test) indicated above the bars. ns. not significant. B. Comparison of 9 mM tryptamine methylation by 254C hINMT in the presence of varying [DTT] concentrations.
(TIF)

S5 Fig Test of DMSe as hINMT substrate (-/+) DTT (15mM), 254C vs 254F. A&B. TMSe production from DMSe for 254C and 254F (-/+) DTT. TMSe production was severely reduced for *both* 254C and 254F (-) DTT ([+] DTT faded for reference from Fig 2). C. SDS-PAGE of 254C and 254F incubated with indicated concentration of substrate in the absence (left panel) or presence (right panel) of 15 mM DTT.
(TIF)

S6 Fig FTIR of DMSe. FTIR spectra of DMSe (Sigma-Aldrich). A. DMSe spectra peaks determined for the present study. B. Expansion of spectra region where oxidized DMSe products would occur. C. Table: Spectra peaks determined for the present study (FTIR) matched to previously published cm^-1^ values (referenced) for (upper) DMSe [24, 25] and (lower) oxidized DMSe products dimethyl selenone (DMSeO_2_) and dimethyl selenoxide (DMSeO) [26]. DMSeO_2_ and DMSeO were not found in the spectra (arrows indicate where they would occur).
(TIFF)

S7 Fig Assays of enzymatic activity for H46P hINMT. H46P hINMT SNP demonstrated no enzymatic activity towards the substrates tryptamine or DMSe (+) DTT 15mM.
(TIFF)
