# Correction: Human indole(ethyl)amine-N-methyltransferase (hINMT) catalyzed methylation of tryptamine, dimethylsulfide and dimethylselenide is enhanced under reducing conditions - A comparison between 254C and 254F, two common hINMT variants

**DOI:** 10.1371/journal.pone.0223546

**Published:** 2019-10-01

**Authors:** The PLOS ONE Staff

There are errors in the Funding statement. The publisher apologizes for the errors. The correct Funding statement is as follows: This study was funded by the School of Medicine and Public Health Grant # UWSMPHPHPRJ82KR.

In the Author Contributions, Brian Torres (BT) should be listed as one of the persons who wrote the paper. The publisher apologizes for the error.
