# Supplementary material for: Human indole(ethyl)amine-N-methyltransferase (hINMT) catalyzed methylation of tryptamine, dimethylsulfide and dimethylselenide is enhanced under reducing conditions - A comparison between 254C and 254F, two common hINMT variants
Source: PLoS One. 2019 Jul 16;14(7):e0219664. doi: 10.1371/journal.pone.0219664 (PMC6634407; doi:10.1371/journal.pone.0219664)
Supplement: S1 Fig — (PDF) [file pone.0219664.s001.pdf]

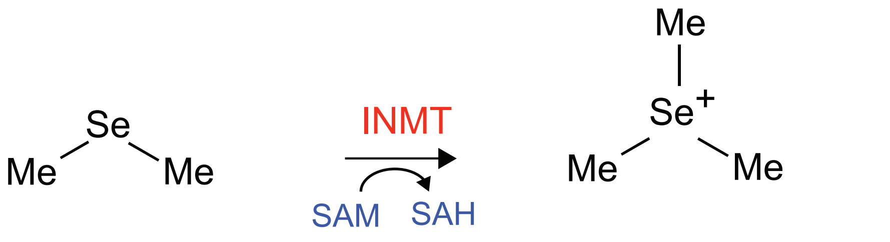

dimethyl
DMSe)

trimethyl selenonium
(TMSe)

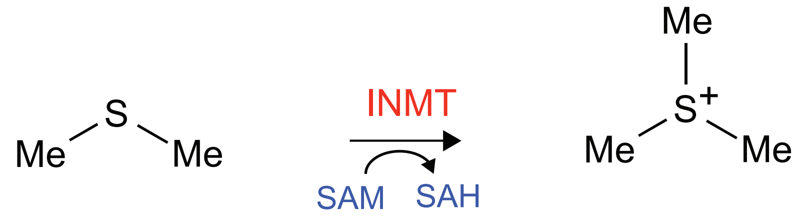

dimethyl sulfide
(DMS)

trimethyl sulfonium
(TMS)

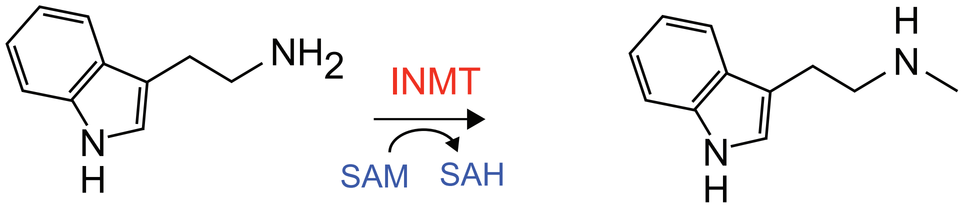

tryptamine

N-methyltryptamine
(NMT)

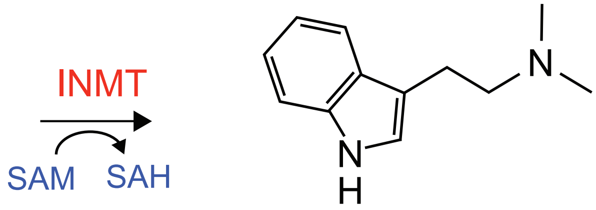

dimethyltryptamine
(DMT)
