# Supplementary figures and images for: Human indole(ethyl)amine-N-methyltransferase (hINMT) catalyzed methylation of tryptamine, dimethylsulfide and dimethylselenide is enhanced under reducing conditions - A comparison between 254C and 254F, two common hINMT variants
Source: PLoS One. 2019 Jul 16;14(7):e0219664. doi: 10.1371/journal.pone.0219664 (PMC6634407; doi:10.1371/journal.pone.0219664)

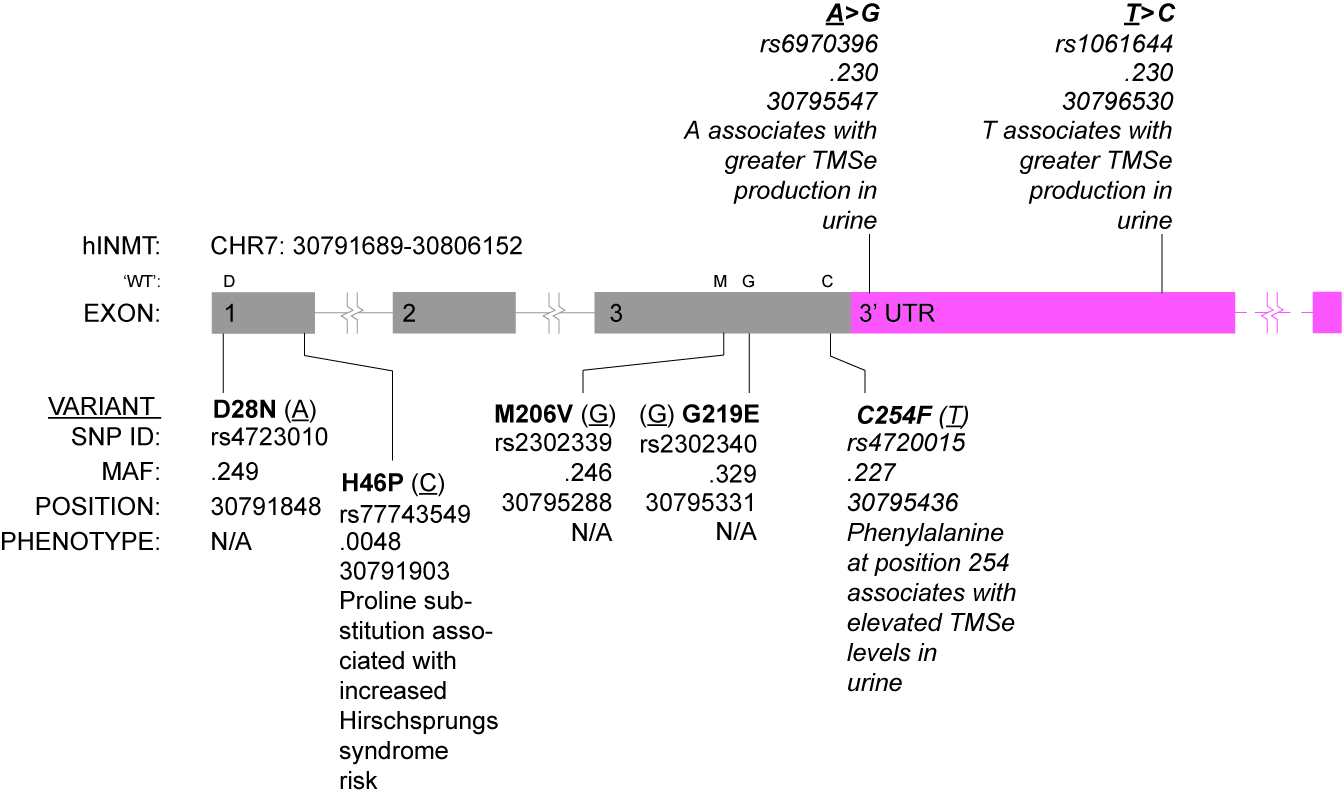

Supplement: S2 Fig — Human INMT gene SNPs referred to in this report. Chromosome number and region are indicated. “WT” refers to the profile of SNPs in a recombinant INMT construct referred to as ‘254C’ in this report. Italicized SNPs are in linkage disequilibrium with each other. SNPs indicate amino acids in the grey exon region and nucleotides in the pink 3’UTR region with the minor allele (according to 1000 Genomes Project [refer to text for details]) underlined and frequency indicated (MAF). Position refers to the chromosomal position on GRCh37.p13. (TIF) [file pone.0219664.s002.tif]

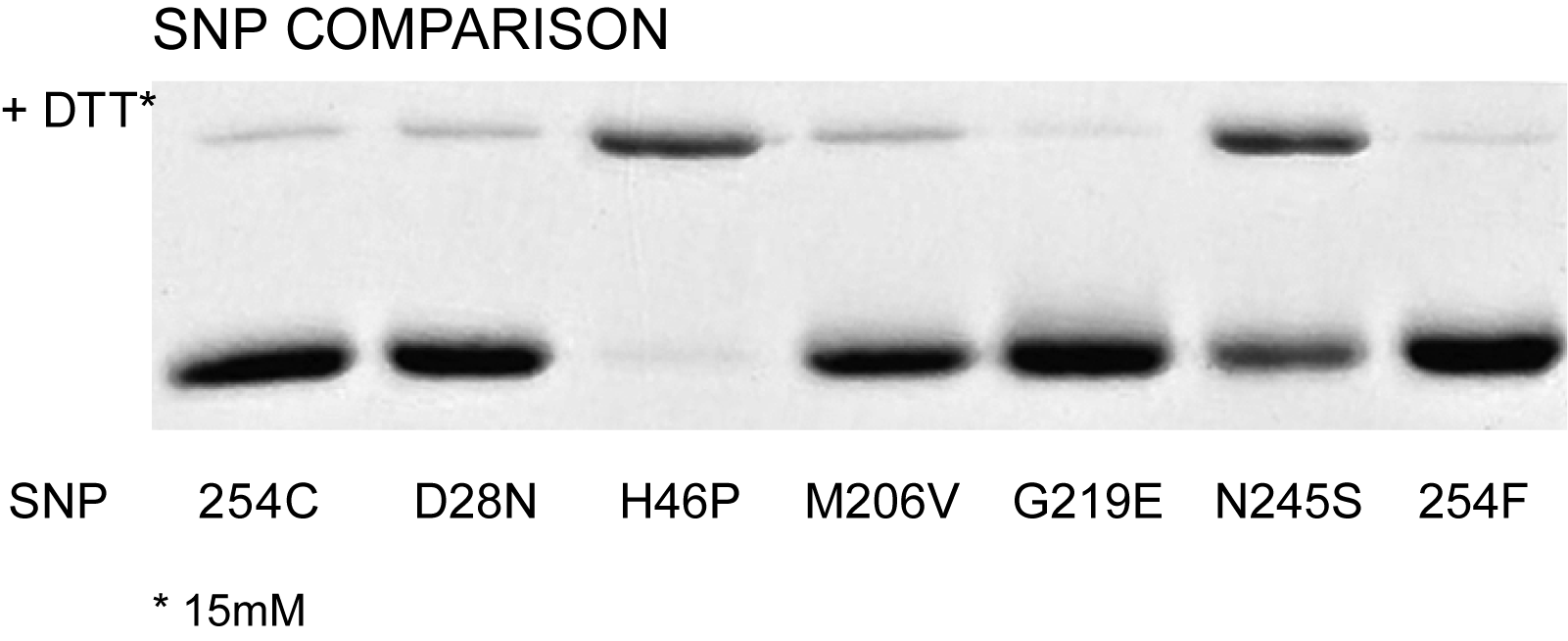

Supplement: S3 Fig — (TIF) [file pone.0219664.s003.tif]

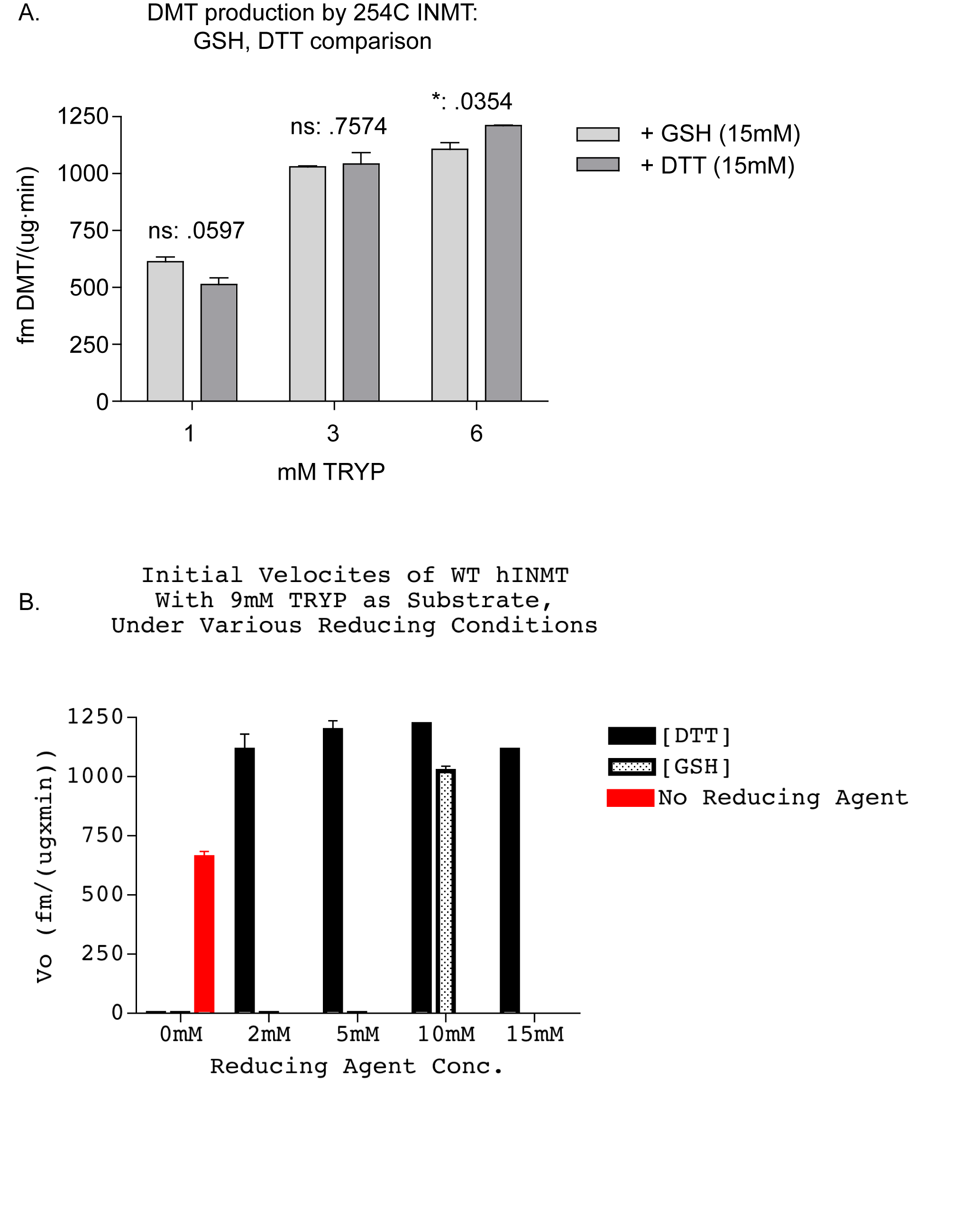

Supplement: S4 Fig — A. Comparison of hINMT-catalyzed methylated of NMT/DMT formation by 254C hINMT for tryptamine in the presence of 15 mM DTT and 15 mM reduced glutathione (GSH). Indicated concentrations of tryptamine were co-incubated with 5 μg of 254C hINMT and 30 μM 14C S-adenosyl-L-methionine as described in Materials and Methods. Each bar represents an experiment performed with duplicate values with p values (two-tailed t-test) indicated above the bars. ns. not significant. B. Comparison of 9 mM tryptamine methylation by 254C hINMT in the presence of varying [DTT] concentrations. (TIF) [file pone.0219664.s004.tif]

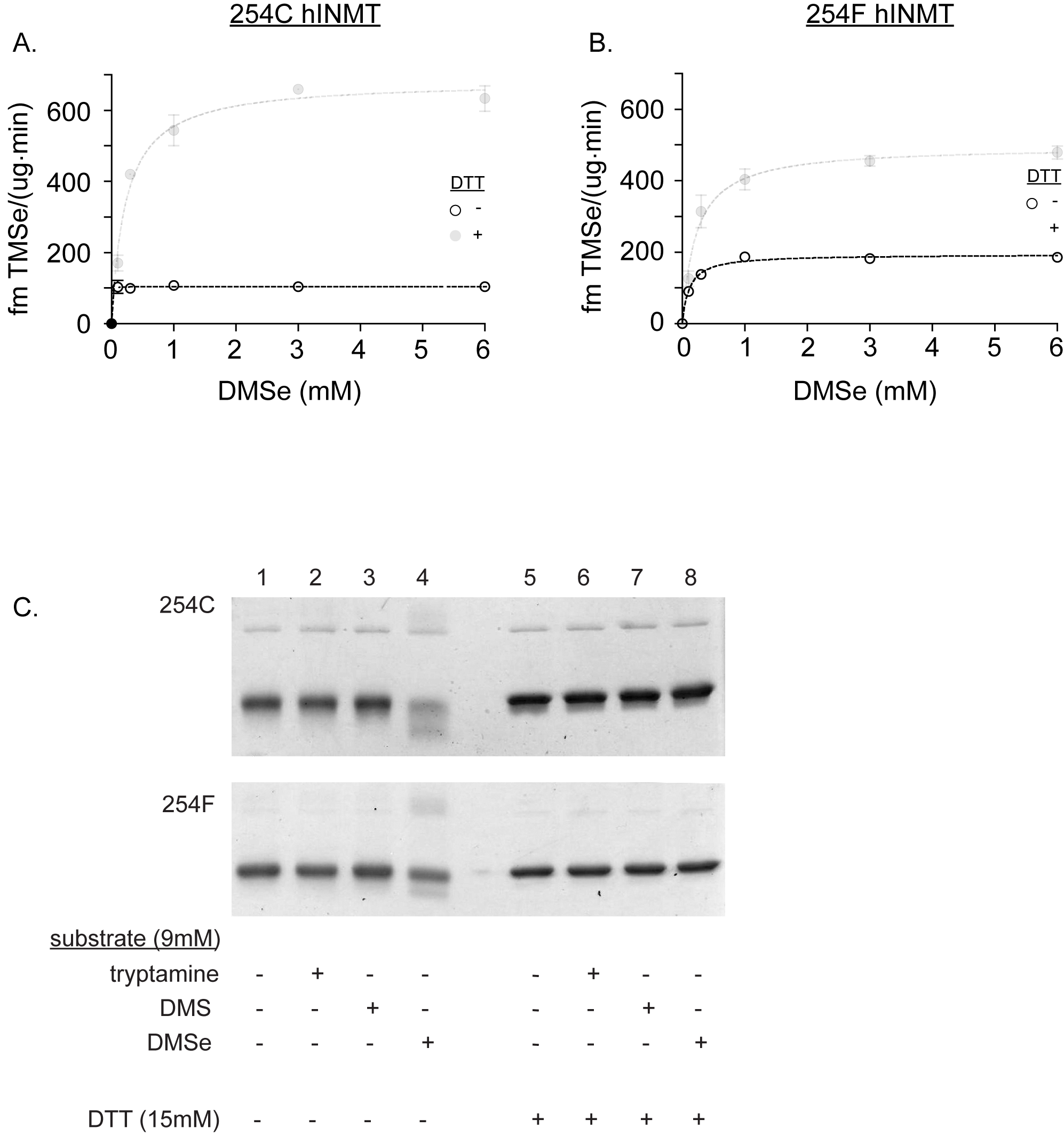

Supplement: S5 Fig — A&B. TMSe production from DMSe for 254C and 254F (-/+) DTT. TMSe production was severely reduced for both 254C and 254F (-) DTT ([+] DTT faded for reference from Fig 2). C. SDS-PAGE of 254C and 254F incubated with indicated concentration of substrate in the absence (left panel) or presence (right panel) of 15 mM DTT. (TIF) [file pone.0219664.s005.tif]

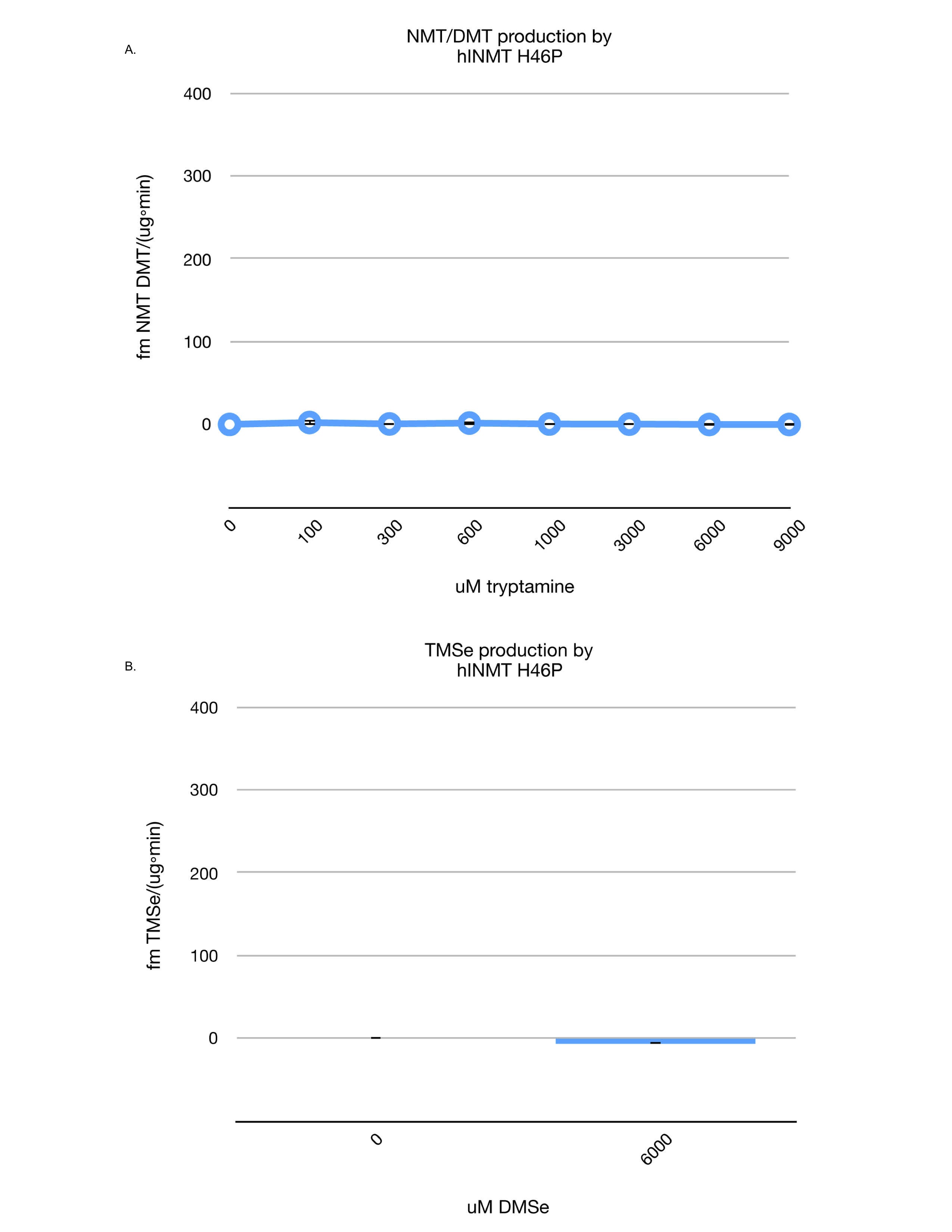

Supplement: S7 Fig — H46P hINMT SNP demonstrated no enzymatic activity towards the substrates tryptamine or DMSe (+) DTT 15mM. (TIFF) [file pone.0219664.s007.tiff]
